# The Antitumour Effect of *Prunella vulgaris* Extract on Thyroid Cancer Cells In Vitro and In Vivo

**DOI:** 10.1155/2021/8869323

**Published:** 2021-01-08

**Authors:** Fangqin Yu, Lele Zhang, Runsheng Ma, Chenguang Liu, Qingduan Wang, Detao Yin

**Affiliations:** ^1^Department of Thyroid Surgery, The First Affiliated Hospital, Zhengzhou University, Zhengzhou, China; ^2^Key Discipline Laboratory of Clinical Medicine Henan, Zhengzhou, China; ^3^Zhengzhou University, Academy of Medical and Pharmaceutical Sciences, Henan Key Laboratory for Pharmacology of Liver Diseases, Zhengzhou, China

## Abstract

*Prunella vulgaris*, a traditional Chinese medicine, has been used to treat various benign and malignant tumours for centuries in China. In our previous studies, *Prunella vulgaris* extract (PVE) was shown to promote apoptosis in differentiated thyroid cancer (DTC) cells. However, whether other mechanisms are involved in the antitumour effect of PVE in thyroid cancer (TC) cells remains unclear. The present study aimed to investigate the antiproliferative and antimigratory effects of PVE on TC cell lines both in vitro and in vivo. First, the TPC-1 and SW579 human TC cell lines were screened by MTT assay for their high level of sensitivity to PVE. Then, the results of cell growth curve and colony formation assay and cell cycle analyses, wound healing, and migration assays demonstrated that PVE inhibited the proliferation and migration of TPC-1 and SW579 cells. Moreover, the antitumour effect of PVE was verified in a subcutaneous xenotransplanted tumour model. Next, MKI67, PCNA, CTNNB1, and CDH1 were screened by qRT-PCR for their significantly differential expression levels in xenograft tissue with and without PVE treatment, and expression of MKI67, PCNA, and CDH1 was verified by Western blot. Finally, an integrated bioinformatics analysis containing protein-protein interaction network, KEGG pathway, and GO analysis was conducted to explore more potential antitumour mechanisms of PVE. In summary, PVE could inhibit the proliferation and migration of TC cells both in vitro and in vivo, which may have been achieved by modulation of the expression of MKI67, PCNA, and CDH1. These data suggest that PVE has the potential to be developed into a new anticancer drug for the treatment of TC.

## 1. Introduction

Cancer of the thyroid gland is the most common endocrine malignancy that has rapidly increased in global incidence in recent years [1]. Differentiated thyroid cancer (DTC), the most prevalent pathological type of thyroid cancer (TC), accounts for approximately 93% of all thyroid cancers [2]. Currently, surgical resection, iodine-131 radiotherapy, and long-term thyroid-stimulating hormone (TSH) suppression remain the three major treatments for DTC [3]. However, surgery might result in various complications, such as hypocalcemia, hoarseness, or postoperative bleeding [4], while iodine-131 treatment and TSH inhibitory therapy have limited efficacy and can cause a series of adverse reactions [5, 6]. Therefore, finding an alternative or adjuvant therapy, such as some herbal and botanical remedies used in oncotherapy, may be of great importance.

Due to their limited side effects and low toxicity, herbal medicines are widely used in not only China but also the United States, where over one-quarter of adults have tried such remedies within the past few years [7]. As a representative traditional herbal medicine, *Prunella vulgaris* and its extracts have been widely used for medicinal purposes for centuries due to their antiviral, antioxidant, anti-inflammatory, and antiallergic functions [8, 9]. More than a thousand years ago, *Prunella vulgaris* extract (PVE) had been used in traditional Chinese medicine to treat sore throat, swelling of the thyroid gland, jaundice, fever, infectious hepatitis, skin allergies, etc. [10, 11]. Today, although many tumour treatments, such as surgery, radiation therapy, and chemotherapy, have been highly developed and are widely accepted internationally, PVE remains an important adjuvant therapy in the clinical treatment of various tumours in China due to its low toxicity and low incidence of adverse reactions [12, 13]. Previous studies identified that PVE can regulate the metastatic microenvironment of tumours via activating multiple signalling pathways and that PVE has antimigratory, anti-invasive, and antiproliferative functions [14]. Because the effective constituents in PVE are complex, some researchers aimed to obtain an ethyl acetate extract from *Prunella vulgaris* (PV) and demonstrate its inhibitory effect on gastric cancer in vitro and in vivo [15]. Moreover, another study found that two polysaccharides isolated from PV could increase the thymus index and spleen index in tumour-bearing mice, suggesting immunomodulatory effects [16]. In addition, publications in recent years have further demonstrated that bioactive chemicals contained in PVE, such as triterpenoids, can obviously inhibit the activity of various malignancies [17].

Our previous studies have proven that PVE induces apoptosis in DTC cells and aimed to reveal the potential molecular mechanism of this effect [18]. However, in our follow-up experiments, we further found that PVE had a significant inhibitory effect on the proliferation and migration of TPC-1 and SW579 cells. In this study, we investigated the antiproliferative and anti-migratory effects of PVE on TC cells (TPC-1 and SW579) both in vitro and in vivo.

## 2. Materials and Methods

### 2.1. Cell Lines and Cell Culture

Human papillary thyroid cancer cell lines (TPC-1, BCPAP), a human squamous thyroid cancer cell line (SW579), and a human follicular thyroid cancer cell line (FTC-133) were purchased from Procell Life Science & Technology Co., Ltd. (Wuhan, China). A normal human thyroid cell line (Nthy-ori 3–1) was purchased from Jennio-Bio Co., Ltd. (Guangzhou, China). All cells were cultured in RPMI 1640 medium (Solarbio, Beijing, China) supplemented with 10% v/v foetal bovine serum (FBS, Gemini) and 1% v/v penicillin/streptomycin (Solarbio) in an atmosphere containing 5% CO_2_ at 37°C. All cell lines used in our experiments have been checked by STR profiling, tested for mycoplasma contamination, and cultured at low passage.

### 2.2. Preparation of PVE

Considering limited knowledge about the bioactive substances in PVE at present, most researchers choose to use crude extracts of PVE to avoid the loss of some important bioactive components [9, 19–21]. As for the extraction protocol, it has been optimized by our research group and Dr. Ding Xiang (who is the pharmaceutical engineer of Guiyang Xintian Pharmaceutical Co., Ltd.) to keep as many bioactive ingredients as possible. PV was provided by Guiyang Xintian Pharmaceutical Co., Ltd. (lot number JG141002; Guizhou, China) and subjected to the following extraction process. The ear of *Prunella vulgaris* (800 g) was extracted three times with a 10-fold volume of 50% ethanol (v/v) at 90°C for 2 h each time. The extract was filtered with gauze two times and then concentrated in a vacuum oven at 60°C to yield a residue (96.8 g). Eight hundred millilitres of distilled water were added to the residue and mixed well. The primary product of PV extraction was finally obtained after incubation at 4°C for 24 h.

For in vitro experiments, the primary product of PV extraction was dissolved in sterile phosphate-buffered saline (PBS) as a stock solution (2.0 g/mL) and then diluted with a complete culture medium (90% v/v RPMI 1640 + 10% v/v FBS) to the desired concentration. For in vivo experiments, the primary product of PV extraction was dissolved in 0.9% w/v sodium chloride.

### 2.3. MTT Assay

Nthy-ori 3–1, TPC-1, FTC-133, SW579, and BCPAP cells were seeded at a density of 5 × 10^4^ cells/well in 96-well plates. After 12 h, the original medium was exchanged with a PVE solution (1 mg/mL) diluted with complete culture medium. Then, they are continued to culture for 24 h, 48 h, and 72 h. And, 20 *μ*L of an MTT (5 mg/mL, Solarbio) solution in PBS was added into each well, respectively, and cells were continually incubated for another 4 h. Then, 150 *μ*L of DMSO (Solarbio) was added to each well, and cells were incubated with shaking at 37°C for 8 min. The absorbance of each well at 490 nm was determined using a Multiskan Spectrum spectrophotometer (BioTek Instruments, USA). Each experiment was performed in triplicate and repeated at least three times.

### 2.4. Cell Growth Curve Determination

A total of 5 × 10^3^ TPC-1 cells/well and 8 × 10^3^ SW579 cells/well were incubated in 24-well plates in the presence or absence of PVE at various concentrations (4, 2, 1, and 0 mg/mL). On days 1–6, the cells in one well of each group were digested, and the cells were counted through trypan blue exclusion. Then, cell growth curves were drawn to describe the change in cell proliferation. Each assay was repeated three times.

### 2.5. Colony Formation Assay

A total of 200 TPC-1 cells and SW579 cells in the logarithmic growth phase were plated in cell culture dishes with a diameter of 6 cm and incubated for 2 weeks in complete culture medium containing PVE (2, 1, 0.5, or 0 mg/mL). Subsequently, cells were fixed with methanol for 20 min and stained with 0.1% w/v crystal violet (Sangon Biotech) for 30 min. The colonies were counted, and all assays were performed in triplicate.

### 2.6. Cell Cycle Analysis

TPC-1 and SW579 cells were incubated for 48 h in a complete culture medium containing PVE at 4, 2, or 0 mg/mL. Then, the cells were harvested, washed with cold PBS, and fixed in 70% v/v ethanol. After incubation for 6–8 h at 4°C, the cells were washed with PBS and suspended in 500 *μ*l of a propidine iodide (PI) solution (Keygen, China) for 30 min before flow cytometry analysis. The percentages of cells in G1, S, and G2 phases were measured by flow cytometry (Beckman, CA, USA). The data were analysed using Multicycle-DNA cell cycle analysis software.

### 2.7. Wound Healing Assay

TPC-1 and SW579 cells were plated in 6-well plates and cultured until they reached confluence. The cell layer was wounded using a sterile tip. Then, a complete culture medium containing PVE at 4, 2, 1, or 0 mg/mL was used for continued cultivation. At 6, 12, and 24 h, wound closure was monitored and photographed using a microscope with a camera system (Olympus, Japan). The migration ability of the cells in each group was calculated by measuring the average scratched area at different time points. These data were analysed using ImageJ software. All assays were carried out in triplicate.

### 2.8. Migration Assay

For the migration assay, 200 *µ*L of TPC-1 or SW-579 cells at a density of 2 × 10^5^ cells/mL in serum-free culture medium containing PVE at 4, 2, 1, or 0 mg/mL was seeded on upper migration chambers (Corning Incorporated Costar, USA) in 24-well plates. Six hundred microlitres of drug-free culture medium containing 10% v/v FBS was added to the lower chamber of each well. After incubation for 36 to 48 h, the cells were fixed with methanol and stained with 0.1% m/v crystal violet. Cells that had not migrated to the upper chamber were gently scraped away using cotton swabs. Then, 33% v/v acetic acid (100 *μ*L) was used to elute crystal violet, and the OD value at 490 nm was detected with a Multiskan Spectrum spectrophotometer (BioTek Instruments), and cells that had migrated to the upper chamber were quantified.

### 2.9. Detection of the Antitumour Activity of PVE In Vivo

Female Balb/*c* nude mice (Vital River, Beijing, China) at 4–5 weeks of age were used to establish a subcutaneous xenotransplanted tumour model. All mice were maintained in a specific pathogen-free (SPF) environment with a 12 h light/dark cycle at 20–25°C with a relative humidity of 40−70% and received freely available sterilized food and water. This study was approved by the Experimental Animal Ethics Committee of Zhengzhou University. Two hundred microliters of TPC-1 cells at a density of 2 × 10^7^ cells/mL in PBS were subcutaneously injected into the mice. When the volume of most tumours had reached 100 mm^3^, the mice were assigned into four groups according to the size of the tumours such that the mean initial tumour sizes in each group were roughly the same. Mice in the three treatment groups were intragastrically administered 0.4 mL of a PVE solution (high-dose group: 500 mg/mL, medium-dose group: 250 mg/mL, low-dose group: 125 mg/mL), while mice in the negative control group were administered 0.4 mL of PBS. Dosage for mice = *X* mg/kg × 60 kg × (0.0026/20 g) (*X*, dosage for human). The dosage given to mice is based on a recommended dosage to human. After conversion by this formula according to body surface area, we finally got a dosage appropriate in mice. Drugs were administered every day for 14 consecutive days. Each mouse was weighed every day, and the tumour volume was measured every two days. Due to experimental animal ethical requirements, the mice were sacrificed by cervical dislocation after CO_2_ euthanasia (flow rate of CO_2_: 5 L/min; size of chamber: 40 × 30 × 25 cm), and the tumours in mice of each treatment group were harvested and weighed. All of the tumour tissue samples were immediately snap-frozen in liquid nitrogen. For histopathological examination, 5 tumour tissue samples from each treatment group were randomly selected, fixed in 4% w/v paraformaldehyde, embedded in paraffin, and finally transferred into a tissue microarray. Then, several 4 *μ*m sections were cut for haematoxylin-eosin (HE) staining.

### 2.10. Quantitative Real-Time PCR (qRT-PCR)

Total RNA was isolated from frozen tumour tissues of mice treated with 500 mg/kg PVE and mice in the negative control group using TRIzol (Solarbio), and cDNA was synthesized with a reverse transcription kit (TaKaRa, Otsu, Japan). qPCR procedure was carried out in a total volume of 20 *μ*l (10 *μ*l Premix, 0.5 *μ*l forward primer, 0.5 *μ*l reverse primer, 2 *μ*l cDNA, 7.0 *μ*l dH_2_O), using SYBR Green PCR reagents (Takara Biotechnology Co., Ltd.) and incubated for 5 min at 95°C, followed by 40 cycles of 95°C for 10 sec, 60°C for 30 sec, 1 cycle of 95°C for 15 sec and 60°C for 1 min. Sequences of the primers used for qPCR are presented in Table S2 (qPCR primers sequences and product size), and the mRNA levels were normalized to those of GAPDH and determined by the 2^−ΔΔCt^ method.

### 2.11. Western Blot Analysis

For Western blot analysis, 50 mg of frozen tumour tissues from mice treated with 500 mg/kg PVE and mice in the negative control group was lysed in RIPA lysis buffer (Leagene, China) containing 1% v/v phenylmethanesulfonyl fluoride (Leagene, China). After quantification by BCA assay, proteins were separated by sodium dodecyl sulfate-polyacrylamide gel electrophoresis (SDS-PAGE) and transferred onto a polyvinylidene fluoride (PVDF) membrane. After blocking with 5% m/v skimmed milk for 2 h, the PVDF membrane was separately incubated with the corresponding antibodies (Table S3: The dilution ratio of antibodies) at 4°C for 12 h. After washing with Tris-buffered saline Tween-20 (TBST) (3 × 10 min), the membrane was incubated with horseradish peroxidase-conjugated secondary anti-rabbit IgG antibody at room temperature for 2 h, followed by visualization using an ECL detection kit (Millipore, Billerica, MA). The Actin protein level was used for normalization. The Imagemaster DVS system was used to determine the relative mean grey values (*A*) of the target product and Actin.

### 2.12. Integrated Bioinformatics Analysis

Protein interactions among MKI67, PCNA, CDH1, and relative proteins were predicted on STRING (http://string-db.org/). KEGG pathway and GO analysis (including biological process, molecular function, cellular component) for these proteins were performed to investigate more potential antitumour mechanisms of PVE.

### 2.13. Statistical Analysis

SPSS 21.0 software was used to analyse the statistical data from the experiments above, and the data obtained in these experiments are expressed as the mean and standard deviation (mean ± SD). Comparisons between groups were performed using one-way analysis of variance. Student's *t*-test was performed to evaluate the significance of differences between mean values. Differences for which *P* < 0.05 were considered statistically significant.

## 3. Results

### 3.1. PVE Inhibited the Proliferation of TC Cells In Vitro

**Figure 1:**
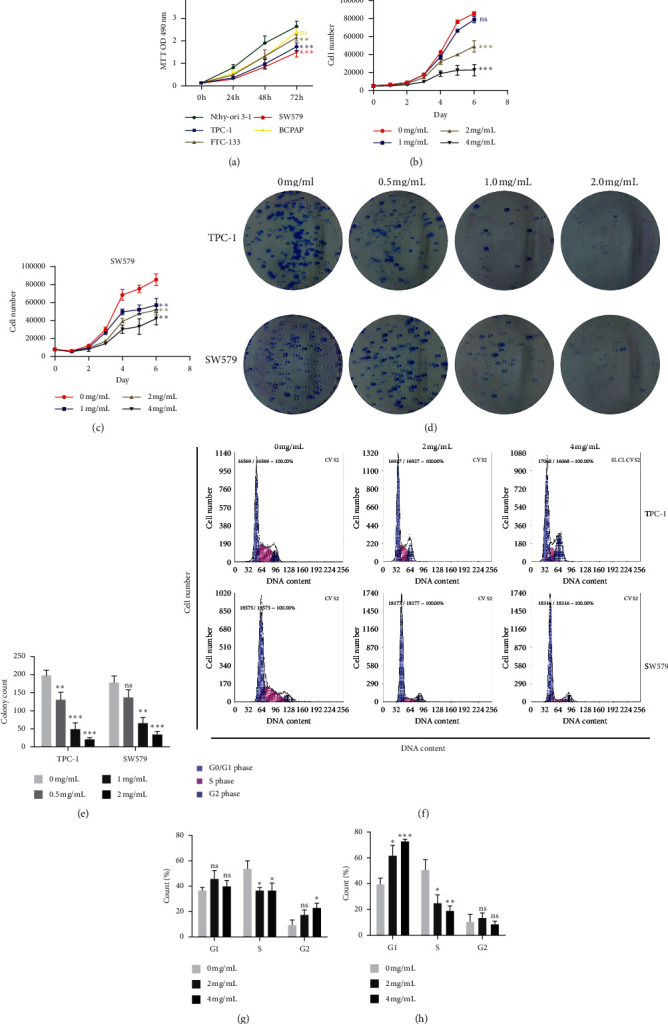
PVE inhibits the proliferation of TC cells. (a) After cultured at complete culture medium containing PVE for 72 (h) the growth of TPC-1 and SW579 cells was significantly inhibited compared to Nthy-ori 3–1 normal thyroid cell line, suggesting that they were more sensitive to PVE treatment than other TC cell lines. ((b), (c)) PVE inhibited the increase of cell number in a dose-dependent manner. ((d), (e)) Colony numbers decreased with increasing PVE concentration. ((f–h)) Cell cycle distribution analysis showed that PVE induced the arrest of both TPC-1 and SW579 cells in the S phase and increased the proportion of SW579 cells in the G0/*G*1 phase. However, the decrease in TPC-1 cells in the G0/*G*1 phase after PVE treatment was not statistically significant. All data are presented as the mean ± SD. One-way ANOVA was performed to identify statistically significant differences (^*∗*^*P* < 0.05, ^*∗∗*^*P* < 0.01, and ^*∗∗∗*^*P* < 0.001).

As a preliminary experiment, an MTT assay was conducted to determine the selective toxicity of PVE in TC cells (Figure 1(a)). Among the four human TC cell lines examined, the TPC-1 papillary thyroid cancer cell line and the SW579 squamous thyroid cancer cell line were finally selected for our subsequent experiments due to their high sensitivity to PVE. The half maximal inhibitory concentration (IC_50_) values calculated from the inhibition ratios of PVE against TPC-1 and SW579 cells (Table S1: Inhibitory effect of PVE at different concentrations on TPC-1 and SW579 cells at 48 h) were 9.294 mg/mL and 6.669 mg/mL, respectively. PVE solutions used in our subsequent in vitro experiments were selected according to the corresponding IC_50_ value.

To evaluate the antiproliferative activity of PVE from different perspectives, cell growth curve, colony formation assay, and cell cycle analyses were conducted. First, the cell growth curves showed that PVE inhibited the growth of TPC-1 and SW579 cells in a dose-dependent manner (Figures 1(b) and 1(c)). To further confirm this finding, we conducted a colony formation assay and found that the colony numbers decreased with increasing PVE concentration (Figures 1(d) and 1(e)). Moreover, we also examined the effect of PVE treatment on cell cycle distribution using flow cytometry (Figures 1(f), 1(g), and 1(h)). PVE induced the arrest of both TPC-1 and SW579 cells in the S phase and increased the proportion of PVE-treated SW579 cells in the G0/*G*1 phase compared to control SW579 cells in the G0/*G*1 phase. However, the decrease in TPC-1 cells after PVE treatment was not statistically significant. Taken together, these results indicate that PVE at a specific range of concentrations inhibited the proliferation of TPC-1 and SW579 cells.

### 3.2. PVE Inhibited the Migration of TPC-1 and SW579 Cells In Vitro

**Figure 2:**
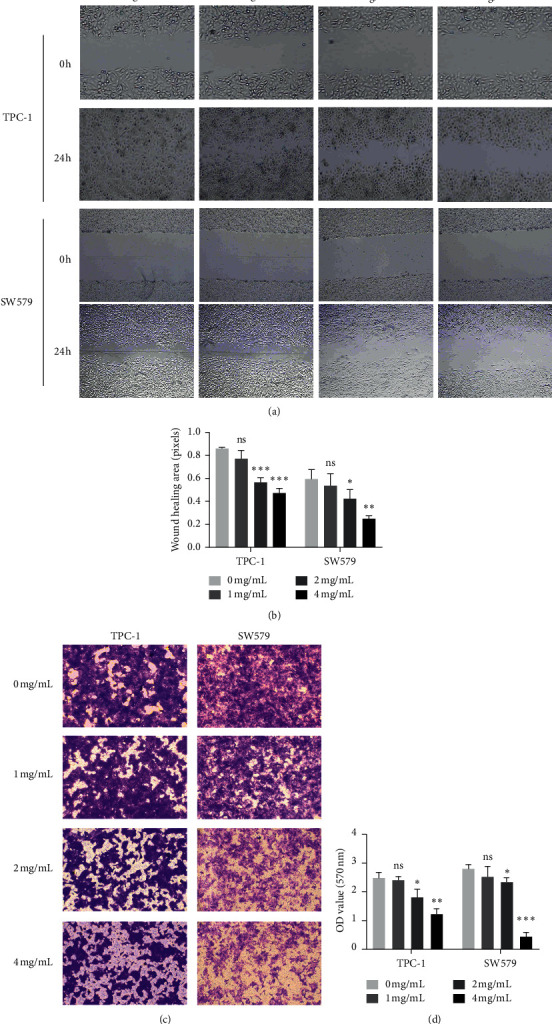
PVE inhibits the migration of TPC-1 and SW579 cells. ((a), (b)) Wound healing ability was significantly decreased in both TPC-1 and SW579 cells after PVE treatment. ((c), (d)) Transwell assays illustrated that PVE impaired the migratory ability of TPC-1 and SW579 cells, and the number of cells that migrated to the lower chamber was represented by the OD570 value and quantified. All data are presented as the mean ± SD. One-way ANOVA was performed to identify statistically significant differences (^*∗*^*P* < 0.05, ^*∗∗*^*P* < 0.01, and ^*∗∗∗*^*P* < 0.001).

The antimigratory effect of PVE on TPC-1 and SW579 cells was examined by wound healing and transwell assays. The wound healing ability (migratory ability) was significantly decreased in both TPC-1 and SW579 cells after culture in a complete medium containing PVE (4, 2, 1 mg/mL) compared with cells in the corresponding negative control groups (0 mg/mL) (Figures 2(a) and 2(b)). Additionally, cell migration was impaired in these two cell lines after PVE treatment, and the number of cells that crossed the chamber decreased with increasing PVE concentration (Figures 2(c) and 2(d)). These results proved that PVE inhibited the migration of TPC-1 and SW579 cells.

### 3.3. PVE Inhibited Tumour Growth in a Subcutaneous Xenotransplanted Tumour Model

To evaluate the antitumour activity of PVE in vivo, Balb/*c* nude mice were subcutaneously injected with TPC-1 cells to construct a xenotransplanted tumour model. Before we performed the formal in vivo experiment, the tumorigenic ability of SW579 and TPC-1 was tested firstly. None of the three nude mice inoculated subcutaneously with different concentrations of SW579 cells (2 × 10^7^/mL, 1 × 10^7^/mL, 0.5 × 10^7^/mL) grew tumours, while all of the three nude mice inoculated subcutaneously with different concentrations of TPC-1 cells (2 × 10^7^/mL, 1 × 10^7^/mL, 0.5 × 10^7^/mL) grew tumours, and their survival time was all longer than 14 days (Figure S1). Finally, TPC-1 cells at a concentration of 2 × 10^7^/mL were selected for our formal experiment in order to achieve a more stable tumorigenesis rate.

First, 28 Balb/*c* nude mice were injected with the same number of TPC-1 cells and randomly divided into four groups when the volume of most tumours was >100 mm^3^. Then, 500 mg/mL, 250 mg/mL, and 125 mg/mL PVE solution (diluted in PBS) were administered as an intervention by gavage. The same volume of PBS was administered by gavage as a negative control (0 mg/mL). During the administration period, a mouse died of asphyxia caused by reflux of the gastric contents after gavage.

**Figure 3:**
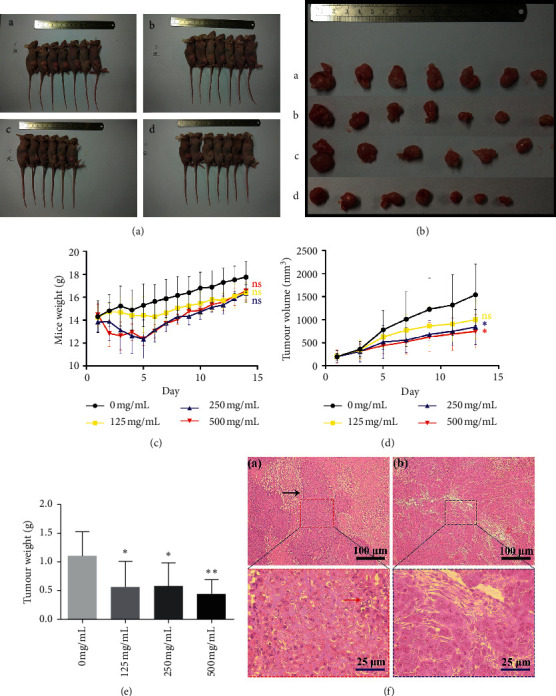
PVE inhibited tumour growth in a subcutaneous xenotransplanted tumour model. ((a), (b)) Macroscopic appearance of mice and tumours in different treatment groups after 2 weeks of PVE treatment (*n* = 7/group). Group a 0 mg/mL; group b 125 mg/mL; group c 250 mg/mL; group d 500 mg/mL. During the administration period, a mouse in group c died of asphyxia after gavage on the first day of administration. (c) Changes in mouse body weight at different groups were not statistically significant. (d) Tumour volumes of mice at groups c and d after 14-day administration presented a remarkable decline compared to group a. (e) Tumour weights of mice at PVE treatment groups (b, c, d) presented a significant decrease compared to group a. (f) HE staining was used to detect the histopathological features of tumour tissues. Upper pictures presented tumour tissues at low magnification, while bottom pictures presented corresponding magnified views. The black arrow points to infiltrating tumour parenchyma, while the red arrow points to tumour cells showing typically disordered mitosis. All data are presented as the mean ± SD. One-way ANOVA was performed to identify statistically significant differences (^*∗*^*P* < 0.05 and ^*∗∗*^*P* < 0.01).

All mice were sacrificed after 14 days of drug intervention (Figure 3(a)), and their tumours were dissected and weighed (Figure 3(b)). Our results showed that the effect of PVE on the mice weight was within an acceptable range (Figure 3(c)), and the volume and weight of tumours were significantly lower in mice administered PVE at a concentration of 500 mg/mL by gavage compared with mice in the negative control group (0 mg/mL) (Figure 3(d) and 3(e)). Haematoxylin-eosin (HE) staining was used to detect histopathological features of the tumour tissues. Infiltrating tumour parenchyma and loosely organized intercellular stroma were seen at 100× magnification, and tumour cells with typically disordered mitosis were seen at 400× magnification (Figure 3(f)). These results suggested that PVE had an antitumour effect in vivo.

### 3.4. PVE Suppresses Thyroid Cancer Progression via Repressing the Transcription and Translation of MKI67, PCNA, and CDH1

**Figure 4:**
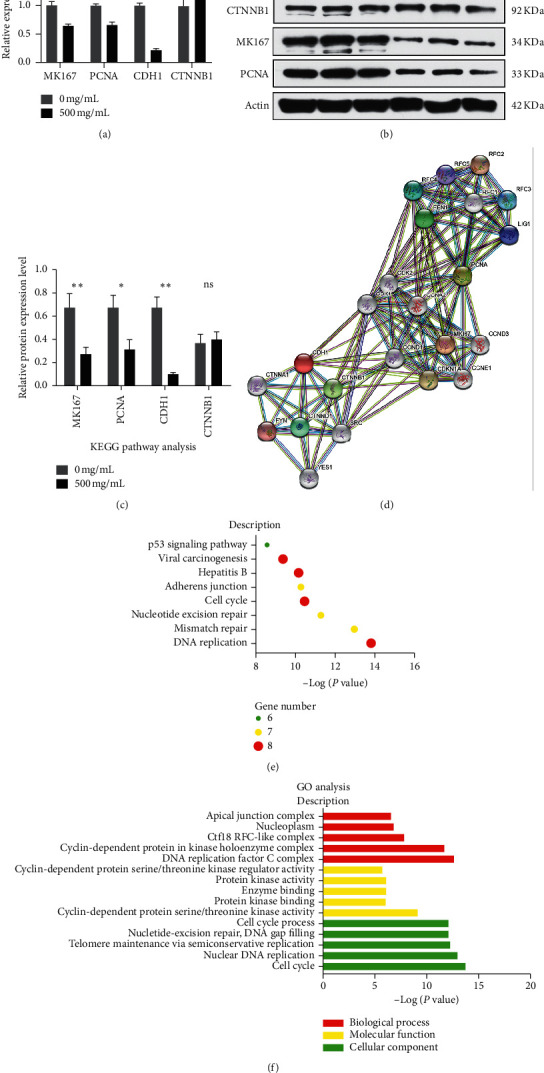
PVE suppresses TC progression via repressing the transcription and translation of MKI67, PCNA, and CDH1. (a) qRT-PCR analysis of xenograft tissue showed that MKI67, PCNA, and CDH1 were significantly downregulated at the transcriptional level after PVE treatment, while CTNNB1 was significantly upregulated. ((b), (c)) Western blot analysis of MKI67, PCNA, CTNNB1, and CDH1 expression in xenograft tissue with and without PVE treatment. (d) The interaction among MKI67, PCNA, CDH1 and relative proteins. ((e), (f)) KEGG pathway and GO analysis for proteins interacting with MKI67, PCNA, CDH1. All data are presented as the mean ± SD. Student's *t*-test was performed to identify statistically significant differences (^*∗*^*P* < 0.05, ^*∗∗*^*P* < 0.01, and ^*∗∗∗*^*P* < 0.001).

To further illuminate the antitumour mechanism of PVE in vivo, the expression levels of 11 common proliferative and migratory molecules (including MKI67, CCND1, PCNA, CHEK1, CHEK2, CDH1, TJP1, VIM, CTNNB1, CD44, SNAIL1) were detected by qRT-PCR in xenograft tissue with and without PVE treatment. MKI67, PCNA, CTNNB1, and CDH1 were finally screened for their significantly different transcription levels with and without PVE treatment (Figure 4(a)). Furthermore, Western blot was used to verify these results at the protein level (Figures 4(b) and 4(c)). To simplify the process of data analysis, only a few xenograft tissues from mice in the negative control group (0 mg/mL) and high-dose group (500 mg/mL) were selected for Western blot analysis. Our results revealed that the protein expression levels of MKI67, PCNA, and CDH1 in the negative control group were significantly higher than those in the high-dose group, while there was no significant difference in the protein expression level of CTNNB1 between the two groups.

Since PVE treatment could affect the expression level of MKI67, PCNA, and CDH1 at both transcription and translation levels, the prediction of protein-protein interaction network focused on MKI67, PCNA, and CDH1 was shown in Figure 4(d). Then, KEGG pathway analysis based on these proteins in the interaction network showed that DNA replication, cell cycle, hepatitis B, and viral carcinogenesis pathways were most likely to be involved during PVE treatment (Figure 4(e)), among which DNA replication and cell cycle corresponded to the results of our proliferative and cell cycle analysis assays. Subsequent GO function analysis further suggested that PVE might exert its antitumour activity by regulating cell proliferation and migration in TC.

## 4. Discussion

The most serious health threats posed by cancers are the malignant proliferation and distant metastasis of cancer cells [22]. As a type of cancer with relatively low malignant potential, the prognosis of TC is better than that of other cancers, but uncontrolled proliferation and the dissolution of basement membranes and the extracellular matrix still occur in the development of TC [23]. At present, we can cure the vast majority of TC cases after early diagnosis in areas with good medical facilities through surgical treatment. However, for some patients with inoperable cancer or with iodine resistance, effective treatments are still lacking. As first-line clinical therapy for DTC, iodine-131 treatment has limited efficacy in some cases with iodine resistance [24], and the efficacy of TSH inhibition therapy requires further medical evidence [25]. Therefore, our study on the antitumour activity of PVE is of great importance and may provide a solution for these patients.

In some experiments, cells need to be treated by PVE for a longer time (such as colony formation assay), so we choose a lower PVE concentration to keep cells in a relatively good condition to support us to finish the experiment. But in other experiments, such as flow cytometry, we only select concentrations below IC_50_ to carry out the experiment. At the beginning of the administration, body weight of mice in middle and high-dose groups showed a slight drop. It is acceptable considering that PVE has a mild gastrointestinal reaction and might cause loss of appetite to some extent. These reasons might lead to some acceptable weight loss.

Recently, remarkable advances have been made in the medication of TC [26]. Among these advances, the inhibition of proliferation and altered migration are the primary antitumour mechanisms [27]. At present, lenvima, sorafenib, caprelsa, cometriq are all the targeted medicine approved by the China Food and Drug Administration for the treatment of recurrent and metastatic and radioiodine-refractory DTC cases [28, 29]. However, the wide variety of side effects and relatively strict indications limit its clinical application.

In our study, PVE showed significant antitumour activity both in vitro and in vivo. Moreover, the preliminary detection of some proliferative and migratory molecules in xenograft tissue revealed the mechanism of the antitumour activity of PVE. The evidence obtained so far suggests that PVE might suppress TC cell proliferation and migration via inhibiting the expression of MKI67 and PCNA and promoting the expression of CDH1. Much evidence is currently available, but much work remains before PVE is developed into a clinical drug. Our first aim should be to determine which compound or compounds in PVE are responsible for its antitumour activity so that we can isolate and purify this compound (s) to conduct further molecular biology research. Some researchers have isolated and purified several triterpenoids from PVE by reverse phase octadecylsilyl (ODS) chromatography, Sephadex LH-20 chromatography, and high-performance liquid chromatography (HPLC) [13, 30], and these triterpenoids have been proven to have significant antiproliferative activity in breast cancer. However, some other scholars suggested that the main anticancer components in PVE are polysaccharides [31, 32]. Due to the heterogeneity of various cancers and differences in purification processes, we may turn to new techniques and analytical methods, such as deep RNA sequencing (RNA-seq) and bioinformatic analysis. Besides, it is well known that cell lines do not always reflect the characteristics of endogenous tumours in patients. Therefore these results still need to be verified in more TC cell lines and TC tissue samples. This is also a limitation that we need to work on further.

## 5. Conclusion

In summary, the present study demonstrates that PVE could inhibit the proliferation and migration of the TPC-1 and SW579 cell lines and that PVE has potential for development into a new anticancer drug for the treatment of TC.

## Data Availability

The data used to support the findings of this study are included within the article.

## Abbreviations

PVE: *Prunella vulgaris* extract
DTC: Differentiated thyroid cancer
TC: Thyroid cancer
TSH: Thyroid stimulating hormone
PV: *Prunella vulgaris*
qRT-PCR: Quantitative real-time PCR
HE: Haematoxylin-eosin
HPLC: High-performance liquid chromatography.

## Supplementary Materials

Supplementary Materials Figure S1: the establishment of a xenotransplanted tumour model in our preexperiment. TPC-1 cells were inoculated at concentrations of 2 × 10^7^/mL, 1 × 10^7^/mL, and 0.5 × 10^7^/mL (from left to right in upper pictures). Macroscopic appearance of mice and tumours at the end of day 14 were shown in bottom pictures; Table S1: inhibitory effect of PVE at different concentrations on TPC-1 and SW579 cells at 48 h; Table S2: qPCR primers sequences and product size; Table S3: the dilution ratio of antibodies.
