# Supplementary material for: The Antitumour Effect of Prunella vulgaris Extract on Thyroid Cancer Cells In Vitro and In Vivo
Source: Evid Based Complement Alternat Med. 2021 Jan 8;2021:8869323. doi: 10.1155/2021/8869323 (PMC7811421; doi:10.1155/2021/8869323)
Supplement: Supplementary Materials — Figure S1: the establishment of a xenotransplanted tumour model in our preexperiment. TPC-1 cells were inoculated at concentrations of 2 × 10^7^/mL, 1 × 10^7^/mL, and 0.5 × 10^7^/mL (from left to right in upper pictures). Macroscopic appearance of mice and tumours at the end of day 14 were shown in bottom pictures; Table S1: inhibitory effect of PVE at different concentrations on TPC-1 and SW579 cells at 48 h; Table S2: qPCR primers sequences and product size; Table S3: the dilution ratio of antibodies. [file 8869323.f1.zip › 8869323.f1/Fig S1.docx]

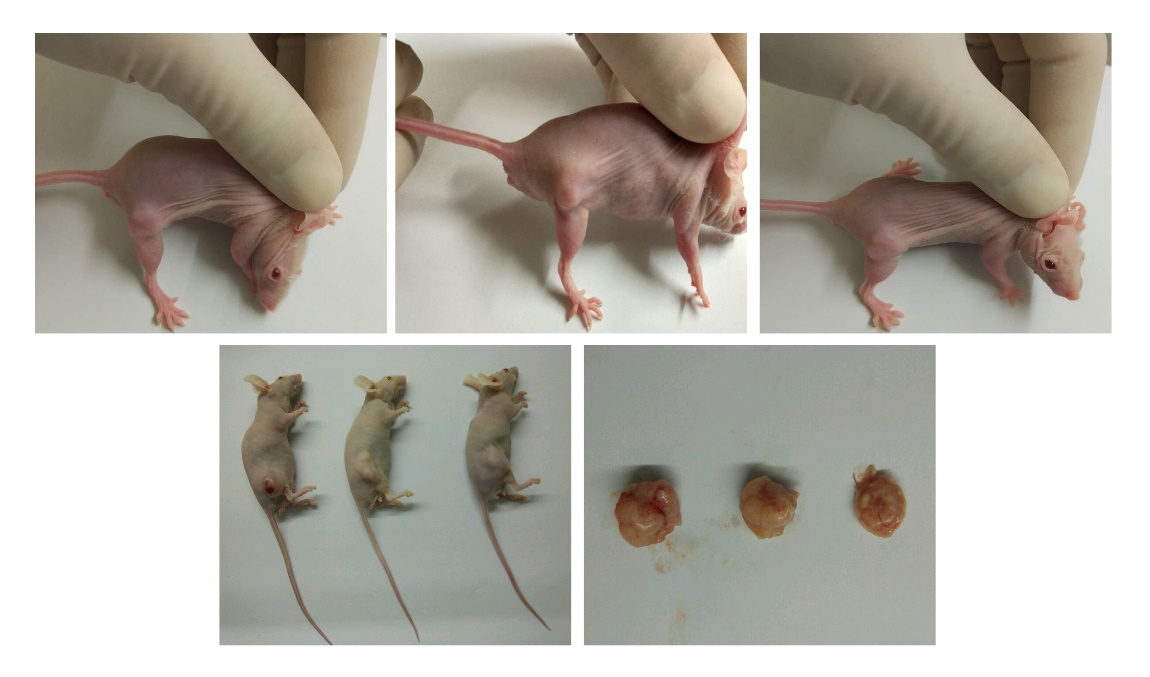


Fig S1 The establishment of xenotransplanted tumour model in our pre-experiment. TPC-1 cells were inoculated at concentrations of 2×10^7^/mL, 1×10^7^/mL, and 0.5×10^7^/mL (from left to right in upper pictures). Macroscopic appearance of mice and tumours at the end of day 14 were shown in bottom pictures.
