# Supplementary material for: The Antitumour Effect of Prunella vulgaris Extract on Thyroid Cancer Cells In Vitro and In Vivo
Source: Evid Based Complement Alternat Med. 2021 Jan 8;2021:8869323. doi: 10.1155/2021/8869323 (PMC7811421; doi:10.1155/2021/8869323)
Supplement: Supplementary Materials — Figure S1: the establishment of a xenotransplanted tumour model in our preexperiment. TPC-1 cells were inoculated at concentrations of 2 × 10^7^/mL, 1 × 10^7^/mL, and 0.5 × 10^7^/mL (from left to right in upper pictures). Macroscopic appearance of mice and tumours at the end of day 14 were shown in bottom pictures; Table S1: inhibitory effect of PVE at different concentrations on TPC-1 and SW579 cells at 48 h; Table S2: qPCR primers sequences and product size; Table S3: the dilution ratio of antibodies. [file 8869323.f1.zip › 8869323.f1/Table S1.docx]

**Table S1 Inhibitory effect of PVE at different concentrations on TPC‑1 and SW579 cells at 48 h**

| **concentration** | **TPC-1** | | **SW579** | |
| --- | --- | --- | --- | --- |
|  | **A_490_** | **inhibition ratio** | **A_490_** | **inhibition ratio** |
| 16 mg/mL | 0.504±0.090 | 0.678±0.063 | 0.263±0.036 | 0.796±0.028 |
| 8mg/mL | 1.040±0.164 | 0.341±0.022 | 0.642±0.045 | 0.501±0.050 |
| 4mg/mL | 1.132±0.157 | 0.268±0.168 | 0.998±0.101 | 0.227±0.045 |
| 2mg/mL | 1.481±0.135 | 0.056±0.052 | 1.027±0.146 | 0.207±0.049 |
| 1mg/mL | 1.548±0.198 | 0.017±0.028 | 1.210±0.204 | 0.066±0.087 |
| 0mg/mL | 1.577±0.226 |  | 1.291±0.108 |  |

TPC-1, a papillary thyroid cancer cell line; SW579, a squamous thyroid cancer cell line; A_490_, the absorbance at 490 nm; inhibition ratio, the inhibition ratio of PVE on TPC-1 and SW579 cells.
