# Supplementary material for: The Antitumour Effect of Prunella vulgaris Extract on Thyroid Cancer Cells In Vitro and In Vivo
Source: Evid Based Complement Alternat Med. 2021 Jan 8;2021:8869323. doi: 10.1155/2021/8869323 (PMC7811421; doi:10.1155/2021/8869323)
Supplement: Supplementary Materials — Figure S1: the establishment of a xenotransplanted tumour model in our preexperiment. TPC-1 cells were inoculated at concentrations of 2 × 10^7^/mL, 1 × 10^7^/mL, and 0.5 × 10^7^/mL (from left to right in upper pictures). Macroscopic appearance of mice and tumours at the end of day 14 were shown in bottom pictures; Table S1: inhibitory effect of PVE at different concentrations on TPC-1 and SW579 cells at 48 h; Table S2: qPCR primers sequences and product size; Table S3: the dilution ratio of antibodies. [file 8869323.f1.zip › 8869323.f1/Table S2.docx]

**Table S2. qPCR primers sequences and product size**

| **Symbol** | **Sequence (5'→3')** | **Product size (bp)** |
| --- | --- | --- |
| GAPDH | forward: 5'-CAGGAGGCATTGCTGATGAT-3' | 138 |
|  | reverse:5'-GAAGGCTGGGGCTCATTT-3' |  |
| MKI67 | forward: 5' -ATTGAACCTGCGGAAGAGCTGA-3' | 105 |
|  | reverse:5'-GGAGCGCAGGGATATTCCCTTA-3' |  |
| CCND1 | forward: 5'-AGGAACAGAAGTGCGAGGAGG-3' | 192 |
|  | reverse:5'-GGATGGAGTTGTCGGTGTAGATG-3' |  |
| PCNA | forward: 5'-TTGCACGTATATGCCGAGACC-3' | 183 |
|  | reverse:5'-GGTGAACAGGCTCATTCATCTCT-3' |  |
| CHEK1 | forward: 5'-ATCAACTCATGGCAGGGGTG-3' | 295 |
|  | reverse:5'-TGGTCCCATGGCAATTCTCC-3' |  |
| CHEK2 | forward: 5'-CTCGGGAGTCGGATGTTGAG-3' | 129 |
|  | reverse:5'-TGCTGGTAGAGGAGCTGGAT-3' |  |
| CDH1 | forward: 5'-GGCTGGACCGAGAGAGTTTC-3' | 156 |
|  | reverse:5'-CAAAATCCAAGCCCGTGGTG-3' |  |
| TJP1 | forward: 5'-TCACGCAGTTACGAGCAAGT-3' | 102 |
|  | reverse:5'-TGAAGGTATCAGCGGAGGGA-3' |  |
| VIM | forward: 5'-GGACCAGCTAACCAACGACA-3' | 178 |
|  | reverse:5'-AAGGTCAAGACGTGCCAGAG-3' |  |
| CTNNB1 | forward: 5'-ATGACTCGAGCTCAGAGGGT-3' | 197 |
|  | reverse:5'-ATTGCACGTGTGGCAAGTTC-3' |  |
| CD44 | forward: 5'-GGAGCAGCACTTCAGGAGGTTAC-3' | 129 |
|  | reverse:5'-GGAATGTGTCTTGGTCTCTGGTAGC-3' |  |
| SNAIL1 | forward: 5'-AAGATGCACATCCGAAGCCA-3' | 237 |
|  | reverse:5'-CATTCGGGAGAAGGTCCGAG-3' |  |
