# Supplementary material for: The Antitumour Effect of Prunella vulgaris Extract on Thyroid Cancer Cells In Vitro and In Vivo
Source: Evid Based Complement Alternat Med. 2021 Jan 8;2021:8869323. doi: 10.1155/2021/8869323 (PMC7811421; doi:10.1155/2021/8869323)
Supplement: Supplementary Materials — Figure S1: the establishment of a xenotransplanted tumour model in our preexperiment. TPC-1 cells were inoculated at concentrations of 2 × 10^7^/mL, 1 × 10^7^/mL, and 0.5 × 10^7^/mL (from left to right in upper pictures). Macroscopic appearance of mice and tumours at the end of day 14 were shown in bottom pictures; Table S1: inhibitory effect of PVE at different concentrations on TPC-1 and SW579 cells at 48 h; Table S2: qPCR primers sequences and product size; Table S3: the dilution ratio of antibodies. [file 8869323.f1.zip › 8869323.f1/Table S3.docx]

**Table S3. The dilution ratio of antibodies.**

| **Symbol** | **Company** | **Catalog number** | **Immunohistochemistry** | **Western blot** |
| --- | --- | --- | --- | --- |
| MKI67 | Proteintech | 27309-1-AP | 1 :8000 | none |
|  |  | 12615-1-AP | none | 1 :1000 |
| PCNA | Proteintech | 10205-2-AP | 1 :200 | 1 :2000 |
| CDH1 | Proteintech | 20874-1-AP | 1 :500 | 1 :5000 |
| CTNNB1 | Proteintech | 51067-2-AP | 1 :1000 | 1 :5000 |
| ACTIN | Proteintech | 20536-1-AP | none | 1 :2000 |
| IgG H&L (HRP) | Abcam | ab6721 | 1 :1000 | 1 :2000 |
